# 17th NATIONAL COMPUTER SECURITY CONFERENCE Baltimore, MD October 11–14, 1994

**DOI:** 10.6028/jres.100.023

**Published:** 1995

**Authors:** Dennis Gilbert

**Affiliations:** Computer Security Division, National Institute of Standards and Technology, Gaithersburg, MD 20899-0001

## 1. Introduction

Annually, the National Institute of Standards and Technology (NIST), Department of Commerce (DOC), and the National Computer Security Center (NCSC), National Security Agency (NSA), co-sponsor the National Computer Security Conference. The conference, most recently in its 17th edition (NCSC17), is a major event on the computer security conference calendar, bringing attendees together with leaders in the field, who report on their research and share experiences. Reflecting the need to more fully appreciate and practically deal with the major technical and social waves of change that we are experiencing, the theme of this year’s conference was *Communicating our Discipline: Strategies for the Emerging Information Infrastructures.*

A large, diverse national and international audience attended the conference, with approximately 2,000 representing government, industry, and academe. NCSC17 provided a forum for technology interchange among system developers, and an opportunity for computer users to exchange ideas and learn about the latest methodologies to apply current computer and information security technology. Many reported that among the most valuable aspects of the conference was the opportunity for contemporaries to network, share information and experiences, and gain new perspectives through the conference’s many and varied activities.

## 2. Conference Program Highlights

This year, main conference tracks focused on research and development, architecture and standards, applications and integration, management and administration, and tutorials for those new to the computer security field. Each track provided eleven $1\frac{1}{2}$ hour sessions of peer-reviewed (i.e., refereed) papers, panel discussions, and/or presentations on basic subject matter. Another offering, *The Learning Track*, explored a variety of issues concerning information technology (IT) security education, training, awareness and professional development. There were also two special sessions devoted to progress on international harmonization efforts and the Common Criteria project. (See Special Sessions on the Common Criteria, below.)

At another special session, chaired by NIST’s Computer Systems Laboratory Associate Director for Computer Security, F. Lynn McNulty, NIST announced and explained a set of NIST and DOC positions on the availability and applicability of the Digital Signature Standard, highlighting that the standard can now be used without fear of copyright infringement concerns.

The opening plenary session featured an address by Sally Katzen, Administrator of the Office of Management and Budget’s (OMB’s) Office of Information and Regulatory Affairs, the presentation of the Conference’s annual System Security Award, and an address by the awardee, Donn B. Parker of SRI International. Ms. Katzen offered insights about the role of the national information infrastructure (NII) in the changing way government does business and delivers its services. Mr. Parker spoke to the need for the security community to broaden its perspective beyond the traditional emphasis on confidentiality, integrity, and availability. (See Sec. 3.) The closing plenary session offered a lively discussion among a panel of distinguished experts on the subject of *Security, Privacy, and Protection Issues in the Emerging Information Infrastructure.*

### 2.1 Research and Development Track

The *Research and Development Track* traditionally addresses technical R&D efforts, including security models and intrusion detection. (A security model is a set of rules and conditions for controlling a user’s access to information resources. Intrusion detection refers to the tools and techniques for detecting that a computer system has been intruded upon or used in an unauthorized way, so that appropriate remedial action can be taken.) As in past years, intrusion detection was a significant area of interest. In a session chaired by R. Bace, NSA, intrusion detection was examined from the perspectives of design methodologies, a model for pattern matching, and current and future applications of artificial intelligence. (See Sec. 4.)

Two sessions explicitly addressed another traditional area of interest in this track, access control (i.e., the process of limiting access to resources to authorized users, programs, processes, or other networks). A panel session, chaired by H. Feinstein, SETA Corp., looked at the future of role-based access control (RBAC), in terms of structure, mechanisms, the environment in which they operate, and how RBAC differs from the traditional trusted system security model (i.e., Bell-Lapadula).

In another session, chaired by D. Cooper, Unisys, one paper described a specific access control model for achieving separation of duties, and two other papers examined architectures for RBAC and a means of implementing RBAC in a trusted on-line, transaction processing environment.

Another panel, chaired by R. Nelson, Information Systems Security, explored non-traditional strategies for using fuzzy security as a means for building flexibility and functionality into trusted systems from a risk management perspective.

In related sessions, M. Schaefer, Arca Systems, Inc. (Arca), chaired a paper session which looked at models addressing the development of secure database systems, and B. Thuraisingham, MITRE Corp., chaired a panel which focused on the inference problem in these systems.

Other sessions in this track included panel sessions on: *Key Escrowing: Today and Tomorrow*, chaired by M. Smid, NIST; and *The Security Association Management Protocol (SAMP)*, chaired by Maj T. Hewitt, USAF6, NSA, the latter addressing security services for communications. Another session presented papers on security in networks and distributed systems, chaired by D. Schnackenberg, Boeing Defense & Space Group; and still another, chaired by S. Jajodia, George Mason University, offered papers on formal methods and modeling regarding secure systems.

To share the learnings from other IT security forums, a panel chaired by E. Leighninger presented the *Highlights of the New Security Paradigms ‘94 Workshop.* Topics included were: fuzzy patterns in data; a health information architecture; applying formal semantics in multilevel logic databases; and the relationships among communication, information security, and value.

### 2.2 Architecture and Standards Track

The *Architecture and Standards Track*, new to this year’s conference, focused on a variety of architectures and standards that are evolving to deal with emerging technical environments in the federal (DoD and civilian) and private sectors.

One panel, chaired by M. Swanson, NIST, addressed *The Development of Generally Accepted System Security Principles (GSSPs).* GSSPs were among a set of recommendations made in a National Research Council Study Report, *Computers at Risk*, published in 1991. (See Sec. 2.4.) Discussed were the GSSPs that NIST is developing under the auspices of Information Systems Security Association (ISSA) in coordination with OMB and with technical assistance from NSA.

In a paper session chaired by W. Jansen, NIST, attendees learned about three differing approaches to security standards, including a taxonomy for viewing and developing them, the use of graphical displays and symantic networks, and vulnerabilities in the use of random pronounceable password generators.

Two other sessions presented varying perspectives on issues related to national and international security criteria and assurance. A panel session chaired by P. Toth, NIST, included results of two workshops on assurance, and a paper session chaired by G. Wagner, NSA, looked at the development history for related procurement guidance.

A panel session co-chaired by E. Flahavin, NIST, and J. Sachs, Arca, examined new challenges for certification and accreditation (C&A) from a variety of government perspectives, especially in environments where system and product interconnectivity and interoperability are at issue. An international panel, chaired by K. Keus, German Information Security Agency (GISA), Germany, looked at product and system certification from the perspectives of representatives of certification bodies of the European Community. (C&A refers to the evaluation of the technical and non-technical security controls to: determine whether a specified set of security requirements are met; and support an official authorization by an appropriate management approving authority, to place a system employing a prescribed set of safeguards into operational use.)

Three sessions focused on security architectures. A panel session chaired by W. T. Polk, NIST, discussed the evolving Department of Defense (DoD) Goal Security Architecture, which reflects requirements for the support of multiple security policies, distributed information processing, conductivity by common carriers, users with different security attributes, and resources with varying degrees of security protection. A paper session chaired by H. Weiss, SPARTA, Inc., and a panel session co-chaired by R. Schell, Novell, GSA, and B. Dwyer, Hewlett-Packard, DCE, focused on related concerns in networked environments, including prominent industry-sponsored security architectures currently under development.

In a two-session minitrack, chaired by J. Sheldon, USA, DISA/CISS, panelists explored current applications and future directions of multilevel security (MLS) (i.e., security in systems which permit access to those possessing different levels of permission). Included was an overview of the NSA Multilevel Information System Security Initiative (MISSI). (See Sec. 6.)

### 2.3 Applications and Integration Track

The means by which security technology is being applied and how security products are being evaluated and integrated into secure systems was the focus of the *Applications and Integration Track.* Of special interest at this year’s conference were the Internet, the NII, and how to achieve security in these environments. One approach to Internet security is through the use of a “firewall,” (i.e., the use of a computer that is placed between the local area network (LAN) and the wide area network (WAN) or global area network (GAN)). An overflow panel session, chaired by J. Wack, NIST, discussed how firewalls work, security policies that can be implemented by means of firewalls, and comparisons of how different firewall configurations support restricted access. Similarly, *Provisions to Improve Security on the Internet*, chaired by J. David, examined what the Internet has done to promote network security, and what steps can be taken to quickly and easily reduce specific risks. *Can Your Net Work Securely?*, chaired by P. Neumann, SRI, examined issues related to the often occurring situation in which distributed systems need to rely on components whose trustworthiness cannot be assured.

*Operational Security Enhancements*, chaired by D. Dodson, NIST, provided a set of papers which looked at ways to improve security in Unix and C2 DOS/Windows-based personal computers, as well as a hardware device for system/data integrity and malicious code protection. Providing multi-vendor interoperability among security-enhanced and traditional UNIX systems was covered in the *Trusted Systems Interoperability Group* panel, chaired by S. Wisseman, Arca, which looked at the TSIG’s related efforts since 1989.

Complementing the intrusion detection presentations (see Sec. 2.1), *Proven Detection Tools For Intrusion Prevention*, chaired by M. Higgins, DISA/CISS, took the audience through detection scenarios and lessons learned from the operational implementation of tools.

Various aspects of system integration were further addressed in *Putting Trusted Products Together*, chaired by B. Burnham, NSA, including ways to approach analysis partitioning and composition analysis. Acquiring MLS system solutions was further debated among the key players in a panel session chaired by J. Sachs, Arca. Paper session *Security Implementations*, chaired by J. Anderson, J. P. Anderson Co., described a variety of security implementations,including thoseinbattlefield, customer network, and academic computing environments. The latter describing a mechanism that was developed to connect dispersed computing resources to achieve distributed processing while not eliminating local control. Panel session, *NSA Concurrent Systems Security Engineering Support*, chaired by B. Hildreth, NSA, looked at NSA’s Test *&* Evaluation Community Network, which must evolve the capability for simultaneously processing unclassified and classified data while supporting both cleared and uncleared users.

Finally, *Views on Vulnerability*, chaired by R. Wood, NSA, addressed computer system vulnerabilities by looking at: evaluating information in computerized alarm systems; a tool for C&A support in DoD automated information systems (AISs); and using a financial management approach for selecting risk management-based safeguards.

### 2.4 Management and Administration Track

The *Management and Administration Track* concentrated on subjects in the management and administration of the security function and the information systems which they support.

Security in organizations can be improved by learning from the experiences of and modeling programs after those that have robust security programs in place. To this end, an informative and lively session, *Model Information Security Programs*, chaired by R. Owen, Jr., Texas Office of the Attorney General, examined IT security programs from the state, federal, private, and academic sectors, highlighting their similarities and differences in areas such as requirements, security organization structure, security management process, and methods of security awareness.

Continuing to explore the theme of his paper on social psychology and information security that won an Outstanding Paper Award at NCSC16, M. E. Kabay, National Computer Security Assn., chaired *Interdisciplinary Perspectives on INFOSEC.* In this panel, a diverse group of academics and practitioners, presented their thoughts as to how the insights of other disciplines can benefit the practice of IT security. Perspectives included anthropology, military science, ethics, psychology, theology, organizational development, and adult learning theory.

Privacy continued to be a subject of strong interest in this track. A particularly lively discussion took place in *Medical Information Privacy: Current Legislative andStandards Activities*, chaired by M. Schwartz, Summit Medical Systems, Inc., which examined the technical and human issues generated by the currently available technology and practices in the medical arena.

Another major theme in this track concerned computer ethics and computer crime. Among the sessions that directly addressed these areas were: *Ethical Issues in the National Information Infrastructure*, chaired by J. Williams, MITRE Corp., and *Detecting and Deterring Computer Crime*, chaired by J. Holleran, NSA. The former explored broad issues, such as equity vs risk, privacy vs accountability, privacy vs surveillance, and international ramifications. The latter paper session looked at intrusion threats, detection using application profiles, and computer crime deterrence. (See Sec. 4.) A third session, *Computer Crime on the Internet*, chaired by C. Axsmith, Esq., ManTech Strategies Associates, provided many views of these subjects as they apply to the Internet. A fourth session, *Risks and Threats*, chaired by D. Gambel, Northrup Grumman, was geared to better understanding the elements of security threats and improving the assessment of risks.

The importance of process improvements was explored in *Current Issues & Trends in Trusted Product Evaluations*, a panel chaired by K. Bruso, NSA. Emphasis was on significant accomplishments in the area of trusted product evaluations during the past year, with special attention focused on two NSA assessment and evaluation programs.

There is a growing appreciation of the role of the IT security professional in the operation of the AIS function and ensuring business continuity. The panel, *Do You Have the Skills to be a Future INFOSEC Professional?*, chaired by W. Maconachy, DISA/CISS, viewed, from the federal government, private sector, and academe perspectives, the types of skills and individual initiatives needed to keep pace with changing work environments and advancing technologies and management challenges.

Another session, *Real Lessons*, chaired by J. Campbell, NSA, presented the audience with the lessons learned from real-world experiences in implementing security programs. Papers in this session addressed: security awareness in the persuasion of managers; the importance of workable network memorandums of agreement; and independent validation and verification of AISs.

In an exciting conclusion to this track, *Computers at Risk (CAR) Recommendations: Are They Still Valid?*, chaired by H. Tipton, CISSP, Member of the CAR Committee, Member of the GSSP Committee, provided a panel discussion that combined recent historical perspective and the lessons of practical experience. In this session, former members of the CAR committee revisited their recommendations in view of today’s information security environment.

### 2.5 Tutorials and Presentations Track

Each year’s conference features a tutorial track. This popular track provides newcomers to the field and others wishing to acquire or “refresh” basic security subject matter an opportunity to do so.

As in the past, this year’s conference offered a set of tutorials on trusted systems, covering such subjects as: *Trust Concepts*, presented by C. Abzug, Information Resources Management College; *Trusted Networks*, presented by R. K. Bauer, Arca; *Trusted Databases*, presented by G. Smith, Arca; and *System Security Engineering, Certification, and Accreditation*, presented by J. Sachs, Arca, which focused on engineering and assessment issues in integrating MLS solutions using trusted products. C. Abzug additionally presented *Criteria Comparisons*, which focused on the differences and similarities of the national and international criteria of Canada, the United States, and Europe, in terms of value to security engineering, and as foundations for the Common Criteria.

Two tutorials were presented by LtCdr A. Liddie, Royal Navy, Information Resources Management College*—Risk Management* and *Security in the Future.* The former focused on the overall risk management process, and the latter, co-presented with J. Sachs, Arca, looked at IT security and its role with respect to enterprises, applications, and information infrastructures.

Two other tutorials addressed security in specific software environments — *UNIX Security*, presented by E. Schultz, Arca, and *Windows NT Security*, presented by J. Williams, Arca. Another tutorial, *Information System Security Officer’s Challenges*, presented by C. Bressinger, DoD Security Institute, focused on the ongoing protection and accreditation of operational AISs. A concluding panel in this track, *IT Security Resources*, chaired by K. Everhart, NIST, offered attendees an overview of major electronic and nonelectronic sources of information on IT security and a discussion of emerging software standards to disseminate and access security-relevant information resources.

### 2.6 Special Sessions on the Common Criteria

The conference continued to reflect community interest evaluation criteria. In addition to the panel and paper sessions discussed in the *Standards and Architecture Track*, two special sessions were held this year related to international harmonization and the Common Criteria (CC). The CC project refers to the work performed by the U.S. and the European Communities (EC) to develop a common basis for evaluating the ability of products and systems to protect confidentiality of data and provide other security controls. Such evaluations are expected to reduce costs to users and vendors.

In the first special session, *International Harmonization, the Common Criteria—Progress & Status*, chaired by E. Troy, NIST, representatives from the European Commission (UK, France, and Germany), Canada, and the United States discussed the CC project, schedules, documents used as input, and the public review process. An overview of the draft Common Criteria document was also presented.

In the second special session, *Security Requirements for Distributed Systems*, chaired by R. Dobry, NSA, panelists from NIST, NSA, the University of Maryland, and the Institute of Defense Analysis identified requirements for providing security for distributed systems and how they saw their efforts relating to the Common Criteria.

### 2.7 The Learning Track

Another feature of this year’s conference was *The Learning Track.* Meaningful security education, training, and awareness for all, and the availability of staff who can ensure that appropriate controls are in place, are part of an overall resource management strategy. The track was framed against the backdrop of an environment that is being shaped by both the emergence of the NII and increasing pressures on all to be more productive. There is also a renewed appreciation by public and private sector organizations about the need to cost-effectively protect information systems resources. The sessions focused on several efforts throughout the IT security community relating to learning initiatives and the professional development of security practitioners. The NIST-sponsored Federal Information Systems Security Educators’ Association (FISSEA) and the National Security Telecommunications and Information Systems Security Committee (NSTISSC)-sponsored Information Systems Security Education, Training, and Awareness (ETA) Working Group coordinated the track.

To introduce the track, *Training Challenges of the 90s*, chaired by J. Pohly, CISS/FISSEA Chair, addressed the security demands that the NII will place on the workforce and the security professional, identified the challenges of complying with training mandates, and outlined proposed solutions.

Training standards are seen as one element of the solution equation. In *Proposed New NIST Training Standards*, chaired by D. de Zafra, Public Health Service, a draft developed by FISSEA that is proposed to replace the NIST training guideline, NIST Special Publication 500-172, was discussed.

A number of sessions in the track looked at the tools, resources, and methodologies for developing and delivering IT security training, and reported on related experiences. These sessions included: *Computer Security Resources that Work*, chaired by B. Cuffie, Social Security Administration; *Tools and Methodologies for Delivering Training*, chaired by J. Jelen, Public Health Service; and *Demonstrations on Computer Security Training Tools*, chaired by A. Stramella, National Cryptologic School. The latter focused on computer-based training packages, videos, and interactive learning tools.

Two issues with which those responsible for IT security programs and security education, training, and awareness must continually deal are garnering management support, and relatedly, competing for budget dollars to implement programs. These issues were addressed in *Effective Marketing of the Computer Security Program to Management*, chaired by J. Hash, Social Security Administration, and *Training Events on a Shoestring Budget*, chaired by S. Pitcher, Department of Commerce, as panelists shared their real-world experiences.

Professionalization and certification are increasingly recognized as integral to how the profession grows, nurtures, attracts, and retains IT security practitioners. *Information Systems Professionalism* — *Professional Development and Certification*, co-chaired by R. Koenig and H. Tipton, International Information Systems Security Certification Consortium (ISC)^2^, explored the current status and future directions of several initiatives underway to professionalize the community and certify the computer security professional.

A particularly interesting session in this track was *Adult Learning and Information Systems Security Training*, presented by E. Martin, Organization and Education Consultant. This session continued with the theme that IT security can benefit from the learnings of other disciplines. The session reviewed recent developmentsin methodology that offer more effective ways of teaching adults to use technical skills that also require individual judgement. It drew on the research and experiences of employer-sponsored training to examine lessons learned about methodologies in use, the basic concepts of adult learning, and the ways these principles can be applied to information systems security training. Concepts were demonstrated by means of experiential exercises.

### 2.8 Closing Plenary

The closing plenary featured a distinguished panel addressing *Security, Privacy, and Protection Issues in Emerging Information Infrastructures.* The panel was co-chaired by Professor Anthony Oettinger, Chairman, Program on Information Resources Policy, Harvard University, and Dr. Brian Kahin, Director, Information Infrastructure Project, Science, Technology and Public Policy Program, Harvard University. Other panelists were Robert Lucky, Vice President Applied Research, Bellcore, and Robert Wilson, MCI.

This interesting panel included lively exchanges among panel members and between the audience and the panel. Each panel member started with a brief statement of issues and perspectives. Overlapping topics included technology advances, pending and possible legislation, intellectual property concerns, major stakeholders, market restructuring, the convergence and integration of media and delivery systems, market share and other business concerns, protection of individual privacy and corporate/organizational proprietary information, other security and protection concerns, standards of due care, and global rather than national scope of the problems (i.e., global information infrastructure (GII vs NII)). One interesting comment came from audience member M. Kabay who said that not until insurance companies punished/rewarded those that avoided/embraced standards of appropriate care to protect their information systems, would significant progress be made. He challenged the community to exert the needed pressures to make that happen.

## 3. Outstanding Contributions to the Field

A particularly satisfying event of each year’s conference is the recognition of an individual who has contributed significantly to the computer security community over an extended period of time. The recipient of this year’s Systems Security Award was Donn B. Parker, Senior Management Consultant, SRI, International. Mr. Parker has conducted extensive research on the human and technical factors involving cases, causes, and the prevention of computer crime, and has promoted a philosophy that security must be treated as a “people” problem, in addition to a technical problem. His research and five books have addressed computer crime, ethics, and information security management. He has contributed to many professional organizations in a variety of capacities. An international lecturer and management consultant, he has served leading businesses, the U.S. Congress, state legislatures, and government agencies. He also created SRI’s International Information Integrity Institute (14), which provides services to 60 of the world’s largest corporations.

Mr. Parker joins a distinguished list of previous Systems Security Award winners which includes Stephen Walker, Dr. Willis Ware, James P. Anderson, Dr. Roger Schell, Dr. Walter Tuchman, and Robert Courtney.

## 4. Outstanding Papers and Best Student Papers

Two outstanding paper awards were presented at this year’s conference. One went to Sandford Sherizen, Ph.D., Data Security Systems, Inc., for his paper *Can Computer Crime Be Deterred?.* This is one of the few papers that has addressed the critical questions of whether computer crimes can be deterred and, if so, by what means. The author points out that we tend to emphasize the computer aspects much more than the criminal aspects in the prevention of computer crime. While deterrence is difficult to achieve, information security programs have essentially neglected attempts to make it work. The author reviews the research findings from criminological and legal studies of deterrence and applies these findings to computer crime prevention. Legislative, law enforcement, and organizational changes need to be made to effectively deter computer criminals. The author makes the case for changing the perceptions of employees and outsiders regarding the risks of getting caught in computer crime, as well as the perceived payoffs from such activities.

The other outstanding paper award went to J. Frank, University of California, Davis for his paper *Artificial Intelligence and Intrusion Detection: Current and Future Directions.* Identification of attempted or ongoing attacks on computer systems and networks, or intrusion detection, is a growing concern for users and administrators of these systems, who rely on their secure operations. This concern increases with each new reported Internet attack. Previous approaches “by hand” to intrusion detection systems (IDSs) made it difficult to create robust, real-time systems. The author notes artificial intelligence (AI) techniques can be effectively applied to the problem, and surveys the methods by which this has been done. The difficulty and computational intensity involved with the activities of data reduction and behavior classification are described. Significantly, the author demonstrates how the use of the technique of feature selection can reduce computational overhead and improve classification of network connections.

Each year, the conference committee invites teachers in IT security-related disciplines to submit papers written by students in a degree program who have not been previously published. This year the conference program committee recognized two excellent student papers. The awards, both for papers in the area of intrusion detection, went to N. Puketza, University of California, Davis, (co-author K. Zhang, advisors B. Mukherjee and R. Olsson) for *Testing Intrusion Detection Systems: Design Methodologies and Results from an Early Prototype*, and S. Kumar, Purdue University, (co-author and advisor E. Spafford) for *A Pattern Matching Model for Misuse Intrusion Detection.*

## 5. Awards Ceremony

As in past years, the conference held a joint awards ceremony in which NIST and NCSC honored the vendors who had successfully developed products meeting the standards of their respective organizations. In the case of NIST, its Computer Security Division provides validation services for vendors to use in testing devices for conformance to security standards defined in three Federal Information Processing Standards (FIPS): Data Encryption Standard (DES), Computer Data Authentication, and Key Management Using ANSI X9.17. This year, many of the NIST awards were for the recently permitted software implementations of DES. In the case of NCSC, vendors are recognized who contribute to the availability of trusted products and who thereby expand the range of solutions customers can use to secure their data. The products are placed on the Evaluated Products List (EPL) following a successful evaluation against the Trusted Computer Systems Evaluation Criteria and its interpretations. (For further information, contact 301-975-2920 regarding the NIST awards and 410-859-4371 regarding the NCSC awards.)

## 6. Other Special Sessions and Demonstrations

A number of other special sessions and demonstrations were available to attendees. These are listed in the following sections.

### 6.1 Electronic Groupware Tools to Address IT Security Challenges

Dr. Corey Schou, of Idaho State University (ISU), has developed an electronic group decision support system that has been effectively applied to a wide range of IT security questions, issues, and challenges. This has been demonstrated through a series of DACUM (Design-a-Curriculum) workshops at ISU, the results of which are contributing to the development of security awareness training materials; IT security curricula; a proposal to revise the NIST Training Guidelines (SP 500-172) with a more rigorous conceptual model for security training; a unified body of knowledge for security practitioners; and knowledge, skills and abilities (KSAs) and plans of instruction for various security-related job categories. The consensus among those who have taken part in the DACUMs, is that the technology can be effectively applied to a wider range of IT security questions, issues, and challenges beyond the DACUM arena.

This year, a portable, ten-station version of the system offered attendees the opportunity to participate in demonstrations and to “test drive” the system. They were able to view the results of the DACUM workshops and a large archive of security information developed at ISU to support the DACUMs. Two groups, one from the GSSP Committee and the other revising the NIST Training Guidelines, each reserved a session on the system to collaboratively “brainstorm” about elements of documents being developed. The former used the system to define the meaning of so-called “pervasive principles,” and the latter for learning objectives associated with AIS functional areas throughout the system life cycle.

### 6.2 Demonstrations of Trusted System Interoperability Group (TSIG) MLS Technologies and Multilevel Information System Security Initiative (MISSI) Products

The TSIG offers an open forum for developers of secure networking systems and those who have a shared vision of making open trusted systems a reality. The MISSI is evolving a series of products which, when combined, provide security services for a variety of MLS environments. These vendor demonstrations showed how many different MLS hardware devices and applications are used in stand-alone and integrated, real-world environments.

### 6.3 European Community IT Security Evaluations

The Information Technology Security Evaluation Facilities (ITSEF) in Europe and the European certification bodies reported on the system and security product evaluations being performed under its program, and demonstrated the product evaluation methodology.

### 6.4 Defense Information Systems Agency (DISA)/Center for Information Systems Security (CISS)

CISS, which is jointly-staffed by DISA and NSA, presented displays and demonstrations to showcase services and products that directly support DoD, including demonstrations by the Automated Systems Security Incident Support Team (ASSIST).

### 6.5 Air Force C4 Systems Security Initiatives

Presented were overviews of Air Force system security initiatives, including demonstrations on intrusion detection and risk management.

### 6.6 Intrusion Detection Workshop

This workshop consisted of several short presentations and discussion periods, including progress reports on development projects; experiences; auditing, legal, privacy, and network security issues; intrusion scenarios; new detection techniques; incident response; and intrusion detection systems requirements.

## 7. Other Activities of Interest

Other activities of interest at the conference included NIST and NSA awareness and information booths where a variety of technical and other publications from each organization were available, including NIST Computer Systems Laboratory (CSL) Bulletins and NSA’s Rainbow Series; demonstrations of NSA’s Dockmaster and NIST’s Computer Security Resources Clearinghouse, each of which offers a wide variety of IT security information through dial-in and Internet access; demonstration of a computer-aided instruction course that was developed by NSA to provide basic-level security information; a combined book exhibit representing a selection of leading publishing firms and the latest selections in computer security, presented by Association Book Exhibit; a booth at which the professional organization Information Systems Security Association (ISSA) presented information and recent newsletters, resource guides, and technical publications; and birds-of-a-feather (BOF) rooms which were used by groups to address self-defined areas of interest.

## 8. Next Year’s Conference

Next year’s conference, the 18th in the series, will be named the National Information System Security Conference. NISSC18 will be held October 10–13, 1995 at the Baltimore Convention Center. For further information, contact the NIST Conference Office, (301)975-2775.

## 9. To Obtain the Conference Proceedings

Single copies of the 742-page NCSC17 conference proceedings are available upon request. Please contact NIST CSL Publications at (301)975-2821.

We are considering putting future conference proceedings, plus additional security-related information, on a CDROM, along with appropriate retrieval capability. Our objective is to keep the price at a minimum, but sufficient to cover expenses. For further information, please contact: (301)975-3359.

